# Was it Good for You? Gender Differences in Motives and Emotional Outcomes Following Casual Sex

**DOI:** 10.1007/s12119-022-09946-w

**Published:** 2022-02-11

**Authors:** Billie E. McKeen, Ryan C. Anderson, David A. Mitchell

**Affiliations:** Department of Psychology, College of Healthcare Sciences, James Cook University, 1 University Drive, Douglas, Townsville, QLD 4814 Australia

**Keywords:** Hookup, Casual sex, Gender differences, Sexual motivations, Emotional outcomes

## Abstract

Casual sex, also referred to as a hookup, has been associated with a range of negative emotional outcomes for women, including regret, anxiety, depression and social stigma. However, it has been argued that it is the nature of the sexual motivation, not gender that influences the emotional outcome. This study was designed to ascertain what motivates people to have casual sex, what emotional outcomes follow casual sex and whether there are gender differences among these variables. Seven hundred and one participants (47% men and 52.8% women) completed a 44-item online survey. Gender differences were found for both sexual motivations and emotional outcomes of casual sex, with women generally having more negative emotional outcomes than men. Additionally, a principal components analysis uncovered four reliable principal motivations underlying engagement in casual sex, and three principal emotional outcomes of casual sex. Predictors of negative emotional outcomes included being motivated to regulate negative emotions and to achieve positive emotions. No predictors (apart from being a man) were found for a positive emotional outcome. While the stigma surrounding female sexual agency is diminishing, results generally support the presence of a sexual double-standard which encourages male promiscuity but dissuades female sexual autonomy.

## Introduction

Gender differences in attitudes toward casual sex have been widely studied. It has been reported that between 44 and 75% of young adults between the ages of 18 and 25 have experienced at least one casual sexual encounter within their lives (Flack et al., 2016; Lyons et al., 2014; Maticka-Tyndale et al., 2003). Casual sex, also known as a hookup or one-night stand, can be described as engagement in sexual acts, with the absence of intimacy (Monto & Carey, 2014). Casual sex is a term that is used to describe a range of sexual behaviours, from a ‘once off’ encounter to frequent encounters of sexual intercourse in the absence of a committed relationship. It is important to note that participation in a hookup may be spontaneous and/or the result of impaired decision making, possibly due to alcohol or another external influencer (Townshend et al., 2014).

Throughout this paper, the term ‘sex’ is used to refer to intimate acts from kissing to coital intercourse and ‘gender’ is used to refer to male and female identities, either biological or social. The term ‘hookup’ is used to refer to sexual activity, from a kiss to coital intercourse, outside of a committed relationship. Sexual expression is both rich and varied, and engagement in casual sex is by no means limited to the cisgender community. For logistical reasons the current study will focus on individuals that identify as either male or female, but it draws no distinction based on either sexual orientation or non-binary gender identity. Previous research has examined variation in attitudes toward casual sex based on sexual orientation (Bothe et al., 2018; Fernandez del Rio, 2019) but has largely focused on the cisgender subset of humanity.

The prevalence of casual sex is difficult to measure, as there is typically a reliance on self-report measures; however, research suggests that casual sex is becoming increasingly socially acceptable within Western societies (Farvid & Braun, 2017). The ready availability of contraception in the 1960s led to a sexual revolution. Sexual norms were liberalised and having sex for pleasure became more acceptable. Other factors reported to have led to a sexual paradigm shift include the enhanced availability and use of pornographic material, changes in alcohol consumption and changes in perceived sexual risk—due at least in part to advances in medical technology (Heldman & Wade, 2010). Within the past decade, the development of online dating services have increased opportunities to access a sexual partner (Ranzini & Lutz, 2016; Sumter et al., 2017). The development of geo-locative smartphone applications and online dating websites has made it easier to meet a casual sexual partner, with 78.2% of participants from a sample of 395 (men and women aged between 18 and 34) claiming to have had casual sex with someone they met through a dating website (LeFebvre, 2018). However, whether or not the liberalisation of sexual norms and acceptance of sex outside of committed relationships has net positive outcomes is unclear.

Despite this, there is evidence that young adults are engaging in sexual behaviour less frequently in current times. Although COVID-19 has had the effect of reducing sexual activity (Arafat et al., 2020; Gleason et al., 2021; Lehmiller et al., 2021; Rosenberg et al., 2020), and this trend emerged prior to the COVID-19 pandemic. Ueda et al. (2020) found that from 2000 to 2018 sexual *in*activity increased in the US among men aged 18–24, and among men and women aged 25–34. The authors speculate that while it is unclear what is ultimately driving this trend, there are quite possibly a number of contributing factors such as: changes in sexual norms; stress and busyness of everyday living limiting leisure opportunities (Wellings et al., 2019); and that the supply of entertainment is competing with sexual activity (Twenge et al., 2017).

Evolutionary and social psychology perspectives suggest that women experience more negative psychological consequences following casual sex than men, including regret, anxiety, and decreased overall mental wellbeing (Fisher et al., 2012; Kennair et al., 2018; Townsend & Wasserman, 2011). In addition to psychological sequelae, there are also a range of health implications of casual sex. Clearly pregnancy (and the risk of) is a potential consequence which affects women to a greater extent than men, but there are also a number of sexually transmitted diseases that disparately impact women. Due to economic, biological, and social factors, women are more susceptible to the acquisition of (and often sustain more damage from) diseases such as the human immunodeficiency virus, chlamydia, syphilis, and herpes simplex virus type 2 (Madkan et al., 2006).

It has been suggested that “the nature of motivational pursuits cannot be adequately understood in the abstract, but rather we must take into account the relational context in which one’s needs are pursued” (Cooper et al., 2011, p. 1333). However, while the weight of empirical literature supports the idea that women generally experience worse psychological outcomes following casual sex than men, some research using motivational frameworks has found no significant gender differences in emotional outcomes following casual sex (Paul et al., 2000; Vrangalova & Ong, 2014). These conflicting findings have prompted researchers to investigate what motivates people to have casual sex, what emotional outcomes follow casual sex, and whether there are gender differences among these variables.

### Evolutionary Perspective

Evolutionary psychologists refer to casual sex as short-term mating and claim that gender differences in sexual motivations and behaviour are innate and universal (Buss & Schmitt, 1993). Sexual motivation is a psychological construct that describes the reasons why people pursue sex (Stark et al., 2015). According to Sexual Strategies Theory (SST), men and women have evolved different underlying motivations for engaging in sex (Buss & Schmitt, 1993). This evolutionary theory on human mating was established with findings from a large-scale study, consisting of 10,047 participants across 37 different cultures (Buss, 1989). Participants were asked to rank desirability of characteristics in short-term and long-term partners, and results indicated gender differences that were consistent cross-culturally. Men regarded a higher quantity of short-term partners as highly desirable, whereas women desired the ability for a partner to provide immediate resources and the potential for him to become a long-term partner (Buss & Schmitt, 1993).

Buss and Schmitt (1993) claim that the differences in reproductive benefits can explain why men report a greater desire to engage in short-term mating and report more positive emotional outcomes than women (Pillsworth et al., 2004; Trivers, 1972). Compared to female sex cells sperm are small, motile, and inexpensive to manufacture, thus men can impregnate multiple partners in a short period of time. In contrast, women have a higher obligate investment in the gestation process, and a typically higher investment in the direct child-rearing process, therefore a long-term partner providing support throughout this process enhances the fitness (and survival prospects) of the woman and her offspring (Pillsworth et al., 2004; Trivers, 1972).

Evolutionary psychologists recognise that both men and women can benefit from short-term sexual encounters, however, short-term relationships are considered less advantageous for women because of the risk of conceiving a child without the support of a long-term mate, which can be detrimental to fitness and survival (Trivers, 1972). Negative emotions have been described as an evolved adaptation to deter decision-making that is not beneficial for reproductive success (Dawson & McIntosh, 2006). Regret following short-term mating is an evolved emotional–cognitive response experienced predominantly by women because it is less reproductively advantageous to engage in short-term mating (Dawson & McIntosh, 2006). Kennair et al. (2018) found that in both Norwegian (*N* = 547) and US samples (*N* = 216), more women than men regretted engaging in their most recent casual sex encounter (41% and 50% vs. 26% and 35% respectively). Worry and physical gratification were measured using single items, compromising the internal validity of the scale used to measure these variables. Results indicated worry, disgust, and pressure were predictive of regret, but sexual gratification and self-initiation of sex was associated with less regret. In support of these findings, a large-scale study (*N* = 24,230), found that 46% of women experienced regret after casual sex compared to 23% of men (Galperin et al., 2013). There was also a substantial gender difference, in the opposite direction, for regret experienced for not pursuing an opportunity for sex (43% men, 16% women). Findings support the general idea that men desire short-term mating more than women, and are consistent with an evolutionary perspective. The authors ultimately suggest that women experience more negative emotions following casual sex because of the higher obligatory costs of sexual reproduction they have paid throughout history, and to avoid future decision-making that are not beneficial for reproduction. However, missed sexual opportunities have historically been associated with higher reproductive fitness costs for men than for women, thus regret following sexual inaction is higher for them.

The evolutionary perspective focuses on gender differences as a result of evolved strategies to enhance reproductive success (Galperin et al., 2013), however, this perspective does not adequately explain motivations to engage in sexual acts that are not concerned with reproducing such as same-sex relations and non-penetrative sexual intercourse. Furthermore, the ease of access to contraception in Western societies supports the notion that people are having sex for other reasons (Emmerink et al., 2016). Although environmental conditions are considered to influence the expression of evolved adaptations, the evolutionary perspective alone does not explain individual, social, and cultural variation.

### Social Perspective

Eagly and Wood (1999) claim that gender differences in sexual motives and behaviour originate from social structure. According to Social Structural Theory, gender differences develop from the contrasting roles men and women accommodate in society. Typically, men occupy dominant roles with greater authority and autonomy, in comparison to women who spend less time in paid occupations and perform more domestic duties (Eagly & Wood, 1999). These contrasting roles have led to a gender hierarchy of power and the development of traditional gendered social scripts. Social scripts are less distinct in gender egalitarian societies, whereby rights, responsibilities and opportunities are less limited by defined gender roles, stereotypes, or discrimination (Darmstadt et al., 2019). This has led to liberalised gendered sexual norms in Western societies.

To determine whether levels of gender equality influenced sexual motives, Eagly and Wood (1999) conducted a reanalysis of the data collected by Buss (1989) using the Gender Empowerment Measure (GEM) to determine levels of gender equality among the 37 different regions sampled. The GEM calculates the difference in men’s and women’s income and representation in political and senior economic positions. Equal representations depict greater levels of gender equality (Klasen & Schüler, 2011). Findings indicated that gender differences were attenuated as rates of gender empowerment increased, supporting the contention that societal factors influence sexual motives.

Although gender differences are attenuated in higher gender egalitarian societies, research suggests that men and women are perceived differently for engaging in the same sexual behaviour (Farvid et al., 2017). Most research indicates that heterosexual men report more previous sexual partners than heterosexual women (Fisher et al., 2012; LeFebvre, 2018; Maticka‐Tyndale et al., 2003). It is important to acknowledge that, assuming a 1:1 sex ratio, the reports of heterosexual partners should be roughly the same for men and women, as each new sex partner for a man must also be a new sex partner for a woman. There is an implicit understanding that while it is socially acceptable for a man to be sexually autonomous, a woman’s sexual agency is discouraged (Farvid et al., 2017). This polarised standard can be socially damaging for women, leading to social stigma and condemnation of women who exercise sexual autonomy outside of a committed relationship (Pickel & Gentry, 2017). ‘Slut shaming’ refers to the pejorative action of degrading women presumed to have engaged in sexual behaviour outside of a committed relationship. Internalisation of this sexual inequality has been associated with negative emotional outcomes for women (Armstrong et al., 2014).

Uecker and Martinez (2017) collected data via an online survey over a six-year period from 2005 to 2011. The large sample consisted of 21,549 college students and indicated that more women (77%) than men (53%) experienced regret after having sexual intercourse outside of a committed relationship. A mediation analysis revealed that 34% of the total effect was attributable to lack of sexual enjoyment, 29% due to perceived loss of respect, and 12% to a loss of self-respect. The large sample size provides support for gender differences with results indicating that women experience more negative consequences following sexual intercourse than men. In this study participants were not provided with a definition for what constitutes a hookup, asking participants to use whatever definition is used among friends (Uecker & Martinez, 2017).

### Sexual Motivations and Outcomes

Research using motivational frameworks has reported minimal and non-significant gender differences in emotional outcomes following sex and argues that outcomes are different depending on the individual’s sexual motivation. Motivational theories posit that sex is used strategically to pursue different goals, and that different motivations explain differences in psychological outcome following sex (Vrangalova, 2015).

In contrast to research documenting gender differences, Vrangalova and Ong (2014) found that gender did not moderate wellbeing following casual sex. Levels of sociosexuality (willingness to engage in casual sex) were measured via a nine-item survey and participants responded to items measuring previous casual sex behaviour, and attitudes and desire for casual sex. In a sample of 371 participants, those that scored highly on sociosexuality reported lower levels of anxiety and depression, and higher levels of self-esteem and life satisfaction, suggesting that casual sex can have positive emotional outcomes. The study concluded that there were no long-term negative consequences on psychological wellbeing following casual sex. Additionally, Vrangalova (2015) investigated the influence of gender and motivation on emotional outcome. Using Self-Determination Theory (Deci & Ryan, 2012), it was hypothesised that people engaging in the same behaviour would have different psychological outcomes depending on whether the motivation was autonomous (self-directed), controlled (other-directed) or amotivated (no intention for behaviour). The longitudinal study consisted of a sample of North American college students (*N* = 528) below the age of 24. Participants were asked to complete surveys at two different time intervals, nine months apart. Depression, anxiety, self-esteem and physical symptoms were measured as outcome variables. Results suggest that engagement in casual sex did not have a long-term impact on psychological wellbeing. Motives that were non-autonomous, such as adhering to peer pressure, were associated with poorer self-esteem and increased depression and anxiety in male participants only. Participants that engaged in casual sex for autonomous self-directed reasons, such as to achieve sexual gratification or personal satisfaction, reported significantly greater levels of self-esteem than participants that had no hookups. Vrangalova concluded that casual sex may increase self-esteem and subsequently enhance positive psychological growth, but that non-autonomously motivated ‘genital’ hook-ups were associated with outcomes of poor self-esteem, anxiety, and depression. The study found no significant differences between men and women, supporting the argument that other factors predict emotional outcomes following casual sex. It is important to realize that while findings suggesting no gender differences are noteworthy, they are somewhat anomalous and stand in contrast to an overwhelming weight of empirical evidence. There are indications that portions of Vrangalova’s work may be methodologically problematic. For example, Vrangalova and Ong (2014) statistically equalized the gender differences they found with transformations (centering) leading to null effects.

### The Current Study

Given the inconsistencies in the literature the following hypotheses were generated:

It was hypothesised that there would be gender differences in the *motivations* for engaging in casual sex (1a), and in the *outcomes* of casual sex (1b).

While a number of studies have looked at the various motivations behind engaging in casual sex (Grubbs et al., 2019; McMahan & Olmstead, 2021; Sevi et al., 2018; Vrangalova, 2015), and separately, the outcomes (emotional and otherwise) following casual sex (see Wesche et al., 2020 for a systematic review), far fewer have examined the relationship between these variables.

Exploratory principal component analyses were conducted separately for (a) *motivations* for engaging in casual sex, and (b) *outcomes* of casual sex. It was furthermore hypothesised that the *outcomes* of casual sex will be able to be predicted by the *motivations* for casual sex (2).

## Methodology

### Participants

The current study asked participants to indicate the gender they identify as. Of the 853 that responded to this question, 399 indicated they were male, 448 indicated that that they were female, and a further 6 indicated ‘other’ or ‘prefer not to say’. Of those selecting from the male/female binary, a total of 59 (29 men) indicated that they had not had a hookup experience in the past. A further 27 responded that they were unsure or that they would ‘prefer not to say’ (15 men).

Given that the study was comparing men and women who had hookup experience, after deletion the eventual sample consisted of 701 men (47%; *M* = 32.85 years, *SD* = 10.83 years) and women (52.8%; *M* = 28.63 years, *SD* = 8.44 years) between the ages of 18–82. The sample was predominantly of European decent (66.7%), although an additional 12.0% indicated that they were from North America, and a further 8.9% indicated that they were Asian. The majority of the sample was heterosexual (75.8%), with 15.8% indicating that they were bisexual and 8.4% indicating that they were homosexual. Half of the sample indicated that they were currently in a relationship (49.7%), and a further 43.8% indicated that they were single.

#### Sampling Procedure

Participants were recruited by posting the link to the online survey on social networking sites such as Reddit and Facebook. The survey link was also uploaded onto the research study participation management system, Sona to recruit current JCU students. Other participants were recruited by word-of-mouth. James Cook University (JCU) students enrolled in eligible subjects received credit points for participation in the survey. Other participants were not incentivised to contribute.

### Measures

Previously validated scales were not utilised due to the exploratory nature of the investigation. The survey was designed to incorporate sexual motives from different perspectives. With regards to emotional outcomes, items were included to measure negative, positive and neutral emotional outcomes relating specifically to the casual sex experience.

A 35-item multidimensional survey was developed and hosted on Qualtrics. The term hookup was defined for participants on the information sheet and consent section of the survey as *‘any sexual activity from a kiss to coital intercourse outside of a committed relationship’*. Participants were explicitly asked to relate questions to their most recent hookup experience.

The survey itself was organised into two parts and items were designed to be brief, using informal language to avoid ambiguity and misinterpretation. Part 1 consisted of 22 items regarding motivations to engage in their most recent hookup. Due to the exploratory nature of this investigation, the items were designed to include motives derived from evolutionary, social and motivational frameworks. The items designed to measure motivations from an evolutionary perspective included “*I had a hookup to start a relationship; to end current relationship; did not like current partner; physically attracted to other person; sexual gratification*” (Meston & Buss, 2007). The influence of social factors was measured with items “*I had a hookup to because I felt pressured by others; under the influence of alcohol or other substance*” (Eagly & Wood, 1999; Farvid et al., 2017). From a motivational framework, items were included to measure avoidant motivations “*because I was feeling lonely; to increase self-confidence; unhappy; miserable; irritable*” and approach motivations “*for personal enjoyment; for fun; feel good about myself; to feel loved; seek affection; sexual satisfaction; physical pleasure*” (Cooper et al., 2011; Gray, 1970, 1987). A reliability analysis suggested that this scale had very good internal consistency (*α* = 0.84).

Part three consisted of 13 statements relating to the emotional outcome following participant’s most recent hookup. Items were designed to measure a range of subjective emotional experiences that related to the casual sex experience. Items to measure negative emotional outcome included “*after the hookup, I felt regret; lonely; rejected; unhappy; negative feelings about myself*” (Cooper et al., 2011; Kennair, 2018). Items to measure positive emotional outcome included “*my mood improved, I felt happier, I felt more confident about myself, I felt sexually satisfied, I felt content*” (Cooper et al., 2011). Two items to measure no emotional change included “*after the hookup, I felt the same; my mood did not change*”. A reliability analysis following appropriate item reversal suggested that this scale had very good internal consistency (*α* = 0.84). All 35 items in the multidimensional survey measured participant’s agreement with statements on a 7-point likert scale (1 = *Strongly agree* and 7 = *Strongly disagree*).

### Procedure

Potential participants were presented with a link to an online study. If they chose to click the link participants were directed to the online survey platform, Qualtrics. Initially they were shown information concerning the study, and asked to provide their explicit consent to participate, before being presented with a series of questions asking about their demographic information (age, gender identity, ethnicity etc.).

In the body of the survey participants were directed to consider their most recent hookup experience and then asked a sequence of 22 questions regarding their motivations for engaging in the hookup. They were then asked 13 questions about the emotional outcomes they experienced as the result of this particular hookup. Participants were told that participation would take no longer than 15 min. 95% of participants completed the survey in 13 min or less. The order of the items in part two and three were randomised in an effort to control for order effects.

## Results

### Preliminary Analyses

As age has been previously shown to influence attitudes surrounding casual sex (Le Gall et al., 2002), men and women were initially compared on this dimension. An independent-samples t-test indicated that men were older than women, *t* (619.90) = 5.70, *p* < 0.001, 95% CIs [2.76, 5.67]. Hence this was used a control variable going forth.

### Gender Differences in Motivations

To test the hypothesis that there would be gender differences in sexual motivations a one-way MANCOVA was performed with gender as the independent variable and each of the sexual motivation items as dependent variables. There was a difference in what motivated individuals to engage in their most recent hookup, based on their gender, *F* (22, 520) = 3.10, *p* < 0.001, *η*_*p*_^*2*^ = 0.12. Additionally, age was a significant covariate here, *F* (22, 520) = 2.40, *p* < 0.001, *η*_*p*_^*2*^ = 0.09 Individual item scores are presented in Table 1. Here a lower mean indicates greater agreement with the item.

**Table 1:** M (SD) scores separated by gender for each motivation item

Motivation (‘I had a hookup…’)	Men	Women
*Because* I wanted to start a relationship	4.46 (1.93)	4.30 (2.03)
*Because I wanted to build an emotional connection with someone*	4.17 (1.99)	3.95 (2.03)
*Because I was physically attracted to them*	2.07 (1.39)	2.15 (1.35)
*Because I wanted to feel close to another person*	3.22 (1.79)	3.12 (1.83)
*Because I was seeking affection from another person*	3.03 (1.89)	2.76 (1.86)
**Because I was feeling miserable	5.09 (1.84)	4.52 (2.06)
*Because I was feeling lonely	3.70 (2.02)	3.22 (1.87)
***Because I felt pressured by the other person	5.79 (1.60)	4.97 (2.01)
*Because I was feeling irritable*	5.50 (1.69)	5.36 (1.77)
*Because I felt unhappy*	4.62 (1.97)	4.01 (1.99)
*For personal enjoyment*	1.62 (.99)	1.97 (1.25)
*For fun*	1.68 (1.06)	2.02 (1.28)
**For sexual pleasure	1.51 (1.04)	2.06 (1.35)
***For sexual satisfaction	1.56 (1.01)	2.16 (1.38)
*Because I was not happy in a current relationship*	5.49 (1.92)	5.61 (1.99)
***For sexual gratification	1.75 (1.13)	2.45 (1.53)
*Because I wanted to feel better about myself	3.68 (1.98)	3.25 (1.89)
*To increase my self-confidence*	3.46 (1.99)	3.37 (1.92)
*Because I wanted to feel loved*	4.12 (1.97)	3.79 (2.04)
*Because I wanted to end a current relationship*	6.22 (1.40)	6.23 (1.35)
*Because I was under the influence of alcohol*	4.41 (2.22)	3.76 (2.31)
*Because I did not like my current partner at the time*	5.80 (1.69)	5.88 (1.76)

Lower scores = higher agreement, *p < .05; **p < .01***p < .001

### Gender Differences in Outcomes

An additional one-way MANCOVA was performed with gender as the independent variable and each of the outcome items as dependent variables. There was an overall difference in the outcomes of an individual’s most recent hookup, based on their gender, *F* (13, 507) = 3.28, *p* < 0.001, *η*_*p*_^*2*^ = 0.08. Age was a significant covariate here, *F* (13, 507) = 1.50, *p* < 0.001, *η*_*p*_^*2*^ = 0.04. Individual item scores are presented in Table 2.

**Table 2:** M (SD) scores separated by gender for each outcome item

Outcome (‘After the hookup…’)	Men	Women
***I felt lonely	4.88 (1.91)	4.06 (1.99)
***I felt unhappy	5.10 (1.81)	4.32 (1.98)
***I felt rejected	5.59 (1.64)	4.82 (2.05)
**I felt regretful	4.87 (1.87)	4.29 (2.08)
***I had negative feelings about myself	5.25 (1.83)	4.51 (2.09)
***I felt sexually satisfied	2.71 (1.38)	3.24 (1.71)
***I felt happier	2.88 (1.47)	3.53 (1.65)
**I felt more confident about myself	2.78 (1.57)	3.28 (1.69)
***I felt content	2.94 (1.55)	3.49 (1.63)
***I was concerned about being negatively judged by others	5.13 (1.93)	4.31 (2.12)
***My mood improved	2.95 (1.38)	3.45 (1.65)
*I felt the same*	3.87 (1.50)	3.89 (1.59)
*My emotions did not change*	4.03 (1.78)	4.18 (1.68)

Lower scores = higher agreement, *p < .05; **p < .01; ***p < .001

### Principal Component Analysis of Sexual Motivations

A principal component analysis was performed to determine the underlying structure of the 22-item survey section that assessed sexual motivations. Oblique rotation (direct oblimin) was deemed the preferred rotation method as this allows correlation between factors and has been argued to yield more accurate results (Costello & Osborne, 2005; Tabachnik & Fidell, 2007). The sample size was considered adequate for a reliable factor analysis. The Kaiser–Meyer–Olkin measure of sampling adequacy was 0.86 (greater than the minimum required value of 0.6; Kaiser, 1974). Additionally, Bartlett’s test of sphericity was significant, χ^2^ (231) = 5861.64, *p* < 0.001 (Bartlett, 1954), supporting factorability of the data. Visual observation of the scree plot indicated that five factors should be kept (Cattell, 1966). As can be seen in Table 3, eigenvalues for five factors were greater than one, supporting that these factors should be retained (Tabachnick & Fidell, 2007). The five factors together accounted for 68.54% of variance in data. Factors one to four (labelled *Regulation of Negative Emotions, Achievement of Positive Emotions, Intimacy Seeking, and Unsatisfying Relationship)* indicated strong internal consistency (α > 0.85). The fifth factor (*External* Influence) however, demonstrated poor internal consistency (α = 0.49) and was therefore not used as a predictor in further analyses. Factor scores were computed for the four internally consistent factors for use in further analyses.

**Table 3:** Factor structure for motivations to engage in casual sex (N = 701)

	Factor loadings				
Item	1	2	3	4	5
1. To regulate negative emotions					
I had a hookup because I felt miserable	.826				
I had a hookup because I felt lonely	.792				
I had a hookup because I was unhappy	.843				
I had a hookup to feel better about myself	.825				
I had a hookup because I felt irritable	.562				
I had a hookup to increase self-confidence	.771				
2. To achieve positive emotions					
I had a hookup for physical pleasure		.896			
I had a hookup for sexual relief		.865			
I had a hookup for fun		.784			
I had a hookup for personal enjoyment		.857			
I had a hookup for sexual gratification		.781			
I had a hookup because I was physically attracted		.546			
3. Unsatisfying current relationship					
I had a hookup to end my current relationship			.821		
I had a hookup because of an argument with partner			.912		
I had a hookup because I was in an unsatisfying relationship			.889		
4. Intimacy seeking					
I had a hookup because I wanted to start a relationship				.778	
I had a hookup to build a connection				.867	
I had a hookup to feel close to another				.824	
I had a hookup because I wanted to feel loved				.717	
I had a hookup because I was seeking affection				.692	
5. External influence					
I had a hookup because I was under the influence of alcohol or other substance					.821
I had a hookup because I felt pressured by others					.758
Eigenvalues	6.16	3.88	2.13	1.80	1.11
Percentage variance	27.99	17.65	9.68	8.17	5.05
Coefficient alpha	.889	.864	.853	.874	.486

Factor loadings < .3 are suppressed

### Principal Component Analysis of Emotional Outcomes

To improve interpretation and use a smaller number of dependent variables in the multiple regression model, a principal component analysis with oblique rotation (direct oblimin) was performed on the 13-item survey measuring emotional outcomes. Again, Bartlett’s test of sphericity was significant, χ2 (78) = 3864.54, *p* < 0.001, supporting the factorability of the data (Bartlett, 1954). Visual observation of the scree plot indicated that three factors should be kept (Cattell, 1966). As can be seen in Table 4, eigenvalues were greater than one for three factors, suggesting their retention (Tabachnick & Fidell, 2007). The three factor-solution accounted for 77.16% of the variance in the data. The three subscales have been labelled *Positive Outcomes, Neutral Outcomes, and Negative Outcomes.* This scale demonstrated internal consistency with Cronbach’s alpha > 0.64.

**Table 4:** Factor structure for emotional outcomes following casual sex (N = 701)

	Factor loadings		
Item	1	2	3
1. Negative emotional outcome			
After the hookup, I felt lonely	.859		
After the hookup, I felt unhappy	.915		
After the hookup, I felt rejected	.817		
After the hookup, I felt regretful	.878		
After the hookup, I had negative feelings about myself	.907		
After the hookup, I was concerned about being negatively judged by others	.686		
2. Positive emotional outcome			
After the hookup, I felt sexually satisfied		.846	
After the hookup, I felt happier		.934	
After the hookup, I felt more confident about myself		.828	
After the hookup, I felt content		.884	
After the hookup, my mood improved		.902	
3. Neutral outcome			
After the hookup, I felt the same			− .931
After the hookup, my emotions did not change			− .925
Eigenvalues	6.83	1.92	1.27
Percentage variance	52.57	14.78	9.80
Coefficient alpha	.931	.639	.842

Factor loadings < .3 are suppressed

### Predicting Outcomes

**Fig. 1:**
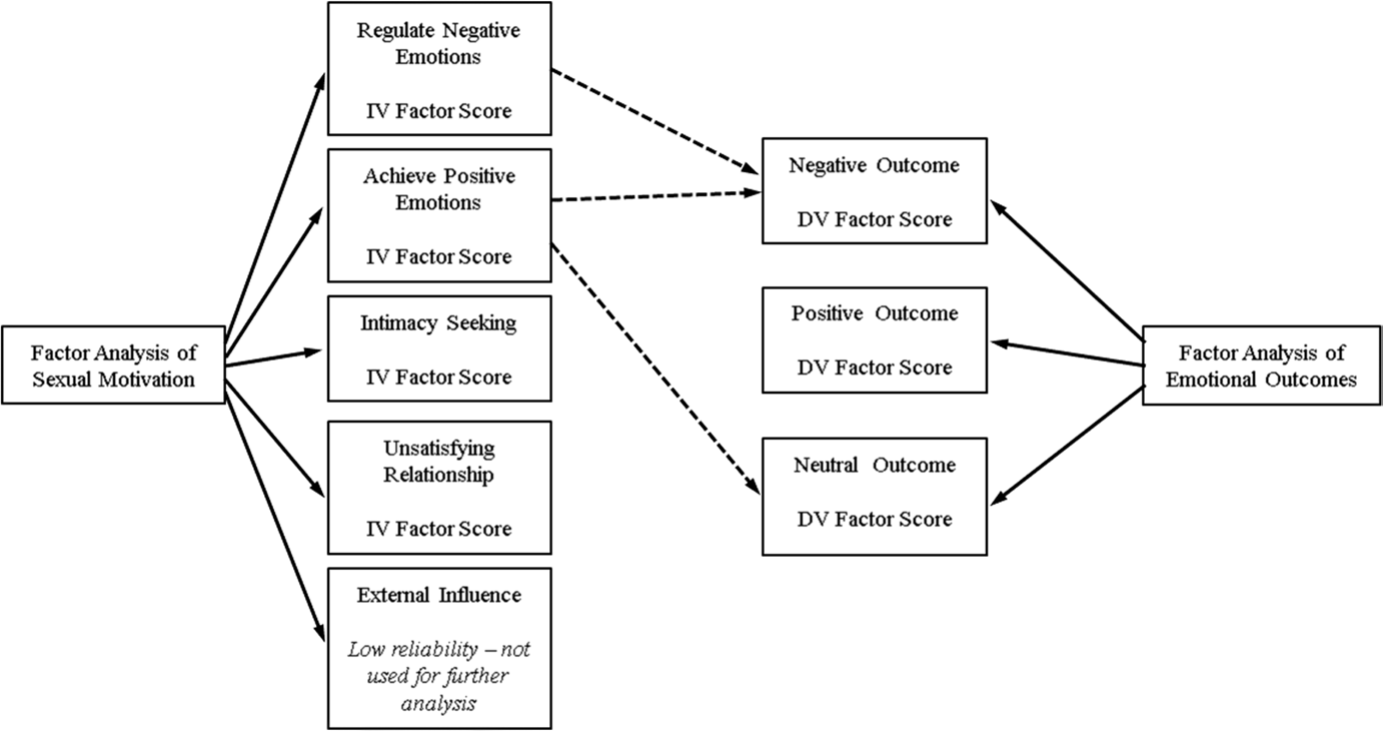
Independent variables and dependent variables used in multiple regression analysis. The dotted line represents the independent variable which predicts the dependent variable

To test the hypothesis that motivation predicts emotional outcome following a hookup, three multiple regression analyses (one for each outcome) were conducted. Factor scores of the sexual motivations were used as independent variables to determine the ability to predict emotional outcomes. Factors scores computed for the three emotional outcomes were used as dependent variables and factor scores computed for motivations were used as independent variables. Figure 1 indicates the independent and dependent variables used in the analyses.

Factor scores were computed for the three factors extracted and used as dependent variables in further analyses.

### Predicting Emotional Outcomes

As indicated in Table 5, *negative* emotional outcomes were predicted by motivations to regulate negative emotions and by motivations to achieve positive emotions, *F* (4, 271) = 6.687, *p* < 0.001). The two motivations collectively accounted for 30% of the variance in scores.

**Table 5:** Multiple regression analysis for sexual motivation predicting negative emotional outcome (n = 276)

Variable	B [95% CI]	$SE_{B}$	$\beta$
Intercept	.103 [− .001 − .206]	.053	
Regulate negative emotions	.206 [.085 − .326]*	.061	.212
Achieve positive emotions	− .206 [− .327 − .085]*	.061	− .195
Unsatisfying relationship	.017 [− .119 − .153]	.069	.014
Intimacy seeking	.023 [− .101 − .146]	.063	.023

*CI* confidence interval, *B* unstandardised regression coefficient, $SE_{B}$ standard error of the coefficient, $\beta$ standardised coefficient, *p = .001.

There were no sexual motivations that predicted *positive* emotional outcomes, *F* (4,271) = 1.455, *p* = 0.216.

As indicated in Table 6, the motivation to achieve positive emotions predicted *neutral* emotional outcomes, *F* (4,271) = 6.262, *p* < 0.001. This motivation accounted for 29.1% of the variance in scores.

**Table 6:** Multiple regression analysis for sexual motivation predicting neutral emotional outcome (n = 276)

Variable	B [95% CI]	$SE_{B}$	$\beta$
Intercept	− .175[− .283 − .067]	.055	
Regulate negative emotions	− .085[− .210 − .040]	.064	− .084
Achieve positive emotions	.294[.168 − .420]*	.064	.268
Unsatisfying relationship	.059[− .083 − .201]	.048	.049
Intimacy seeking	.050[− .079 − .179]	.065	.049

*CI* confidence interval, *B* unstandardised regression coefficient, $SE_{B}$ = standard error of the coefficient, $\beta$ standardised coefficient, *p < .001.

## Discussion

It was hypothesised that there would be gender differences in motivations and emotional outcomes relating to casual sex. These hypotheses were supported, with overall gender differences for both, and a number of strong gender differences for individual items. Furthermore, when reduced to discrete factors, motivations predicted emotional outcomes.

### Gender Differences in Motivations and Outcomes

Although there was an overall gender difference in the motivations for casual sex, it is noteworthy that men and women similarly endorsed statements such as *‘I had a hookup for personal enjoyment/fun’*. Such findings support the idea that social stigma surrounding women’s sexual agency is diminishing. There was also a significant overall gender difference in emotional outcomes following casual sex, and differences for 11 of the 13 individual outcome items. Women reported significantly more negative emotional outcomes than men, including loneliness, unhappiness, rejection, regret, general negative feelings, and a perception of negative judgment from others. Conversely, men reported greater sexual satisfaction, happiness, self-confidence, contentment, and mood improvement. Each of these findings is consistent with the general idea that men experience some kind of emotional enhancement from engaging in casual sex, but for women the emotional effect is reductive. While the statistical effect-size of the gender difference here was reasonably small (*η*_*p*_^*2*^ = 0.08), it is worth noting that of the 11 items indicating a gender difference, women reported a greater agreement to 6, while the reverse was true for five, thus the *average* difference misrepresents the more nuanced story.

Women reported significantly more regret, loneliness, unhappiness, rejection and negative feelings about one’s self in comparison to men following their most recent hookup experience. It is important to note that this finding is consistent with research from an evolutionary perspective, which suggests that women experience more regret than men because short-term sexual relationships are considered less advantageous for women’s reproductive success, and conversely, advantageous for men’s reproductive success (Galperin et al., 2013; Kennair et al., 2018). However, the item that loaded on the same factor as all of these items was *‘concern about being negatively judged by others’* which supports the sexual double standard from a social psychological perspective (Eagly & Wood, 1999).

Within Western culture, women are supposedly empowered and gender equality regulations are in place to enhance equality in opportunities, however, the findings of this investigation suggest that women do not experience casual sex in the same way as men. Women reported more concern about being negatively judged by others after engaging in casual sex than men. There is a risk of social stigma, namely slut shaming leading to social isolation for women, marking them as lower in status and less deserving of respect with the risk of social isolation, poor reputation and negative emotions (Armstrong et al., 2014).

Western culture supports gender equality, and levels of sexual permissiveness are arguably becoming more liberal. Engagement in casual sex is becoming increasingly acceptable, and it is noteworthy that the modest effect sizes in gender differences reported here may suggest that the disparity is decreasing, but the risk of experiencing negative emotional outcomes is still considerably greater for women than it is for men (Armstrong et al., 2014). Men are rarely threatened with social repercussions in the same way that women are, therefore expressing sexual autonomy is arguably less prohibitive for them (Farvid et al., 2017).

While the current study reported considerable gender differences in emotional outcomes, dissimilar findings of a minimal or null effect may be an artefact of methodological inconsistency. Vrangalova (2015) used a sample of young adults enrolled in higher education, however, the current sample was more heterogeneous, and inclusive of adults from more diverse backgrounds with ages ranging from 18 to 82.

### Motivations Predicting Emotional Outcomes

The motivation to regulate negative emotions accounted for most of the variance in the data, suggesting that many individuals engage in casual sex in an effort to regulate their negative emotions. This motivation was also predictive of negative emotional outcomes. Having casual sex to manage feelings of loneliness, misery, unhappiness and irritability may lead to negative emotional outcomes, including feelings of regret, rejection, unhappiness, loneliness, negative feelings towards one’s self, and concern about being negatively judged by others. Although, the non-causal nature of the relationship bears mention, it may just be that a negative mindset is associated with both problematic motivations for and outcomes of casual sex.

We did not find a motivation that predicted positive emotional outcomes. It may simply be that a positive emotional outcome following casual sex is too difficult to reliably predict with only a small (unnuanced) set of variables, or that the more likely outcome of casual sex may be the reduction of something negative as opposed to the addition of something positive. However, the motivation, to achieve positive emotions, was found to predict neutral emotional outcomes. This may suggest that having sex for personal gratification, enjoyment, or fun can lead to an unchanged mood and feelings remaining the same.

### Strengths, Limitations and Recommendations for Future Research

While the theme of gender differences in attitudes toward casual sex is by no means a new one, we believe that the current study is unique in a number of ways. For one, many studies in this area recruit younger (college-aged) samples. The sample of the current study, recruited via social media, was heterogeneous (and hence more generalizable) in terms of both age and ethnicity. Additionally, few studies have previously attempted to quantify the relationship between emotional motivations for and the emotional outcomes of casual sex.

In the current study participants were asked to refer to their most recent hookup experience but were not asked when this experience occurred. Future studies may wish to quantify this in order to determine whether the passage of time has an impact on emotional outcomes (or perception thereof). It is reasonable to suggest that as time passes since one’s last hookup experience, the strength of the emotions associated with the event may be tempered by temporal distance. Future studies may also benefit from measuring an individual’s sociosexual orientation and general wellbeing as both may be important control variables. For example, an individual that exclusively seeks short-term mating opportunities and/or is psychologically unwell is presumably motivated to engage in sexual behaviour for different reasons than a more stable, relationship oriented person.

The current study clearly defined the term ‘hookup’, based on previous research in the area (Napper et al., 2016; Owen et al., 2011). Defining the term for participants was an important component of the study, to ensure that participants were referring to the same range of sexual behaviours. Research on this phenomenon is methodologically inconsistent and often uses vague definitions for casual sex, such as “use whatever term you use with your friends” (Uecker & Martinez, 2017) and “sexual behavior occurring outside of long-term romantic relationships” (Vrangalova, 2015). However, the frequency with which an individual engaged in casual sex was not measured. Future research may wish to do so as emotions associated with a behaviour (especially highly valent ones) may well be enhanced as the frequency of said behaviour increases.

While the current study compared those who identify as male to those who identify as female, it neglected to gather information regarding transgenderism and gender identities beyond the traditional binary. Doing so was consistent with the weight of previous empirical literature (but see Wilson et al., 2010), however, if for no other reason than scientific rigour, further research into transgender/non-binary populations is needed. Future studies may wish to consider stratifying their sample by gender identity in order to gain a more nuanced understanding of attitudes toward, and emotional outcomes of, casual sex.

Finally, although the sample employed in the current study was ethnically heterogeneous, it was predominantly Caucasian. While this is consistent with the overwhelming majority of previous research, racial discourse in this area is critical to the ongoing discussion surrounding casual sex. Future studies should consider sampling from non-Western areas, or potentially stratisfying their sample by race.

The current study makes a unique and meaningful contribution to the literature in that it established that some (but not all) outcomes of casual sex can be predicted based on understanding an individual’s motivations for engaging in such. Namely, people who engage in sex to regulate negative emotions are likely to experience negative emotional outcomes. It is unclear as to whether this is because causal sex enhances pre-existing negative emotions or is just not an effective method for managing such emotions. It may be that the current study was unable to determine predictors of positive emotional outcomes following casual sex simply because we did not ask the right questions. Future studies in this area may consider conducting qualitative interview research in order to gain a richer and more nuanced understanding of this phenomenon, and potentially insight into attitudes and behaviors associated with favourable emotional outcomes.

The takeaway message of this research is clear: when engaging in anything from a kiss to coital intercourse outside of a committed relationship, ensure your underlying motivation is not to regulate negative emotions.

## Data Availability

Data on which this study is based can be found at https://osf.io/35ynj/?view_only=870a2f3e423d44e48366d41e2a7428c2.

## References

[CR1] Arafat SY, Alradie-Mohamed A, Kar SK, Sharma P, Kabir R (2020). Does COVID-19 pandemic affect sexual behaviour? A cross-sectional, cross-national online survey. Psychiatry Research.

[CR2] Armstrong EA, Hamilton LT, Armstrong EM, Seeley JL (2014). “Good girls” gender, social class, and slut discourse on campus. Social Psychology Quarterly.

[CR3] Bartlett MS (1954). A note on the multiplying factors for various chi square approximations. Journal of the Royal Statistical Society.

[CR4] Bőthe B, Bartók R, Tóth-Király I, Reid RC, Griffiths MD, Demetrovics Z, Orosz G (2018). Hypersexuality, gender, and sexual orientation: A large-scale psychometric survey study. Archives of Sexual Behavior.

[CR5] Buss DM (1989). Sex differences in human mate preferences: Evolutionary hypotheses tested in 37 cultures. Behavioral and Brain Sciences.

[CR6] Buss DM, Schmitt DP (1993). Sexual strategies theory: An evolutionary perspective on human mating. Psychological Review.

[CR7] Cattell RB (1966). The scree plot test for the number of factors. Multivariate Behavioral Research.

[CR8] Cooper ML, Barber LL, Zhaoyang R, Talley AE (2011). Motivational pursuits in the context of human sexual relationships. Journal of Personality.

[CR9] Costello AB, Osborne J (2005). Best practices in exploratory factor analysis: Four recommendations for getting the most from your analysis. Practical Assessment, Research, and Evaluation.

[CR10] Darmstadt GL, Heise L, Gupta GR, Henry S, Cislaghi B, Greene ME, Hawkes S, Hay K, Heymann J, Klugman J, Levy JK, Raj A (2019). Why now for a series on gender equality, norms, and health?. The Lancet.

[CR11] Dawson BL, McIntosh WD (2006). Sexual strategies theory and internet personal advertisements. Cyber Psychology & Behavior.

[CR12] Deci EL, Ryan RM (2012). The Oxford handbook of human motivation.

[CR13] Eagly AH, Wood W (1999). The origins of sex differences in human behavior: Evolved dispositions versus social roles. American Psychologist.

[CR14] Emmerink PM, van den Eijnden RJ, Vanwesenbeeck I, ter Bogt TF (2016). The relationship between endorsement of the sexual double standard and sexual cognitions and emotions. Sex Roles.

[CR15] Farvid P, Braun V (2017). Unpacking the “pleasures” and “pains” of heterosexual casual sex: Beyond singular understandings. The Journal of Sex Research.

[CR16] Farvid P, Braun V, Rowney C (2017). ‘No girl wants to be called a slut!’: Women, heterosexual casual sex and the sexual double standard. Journal of Gender Studies.

[CR17] Fernández del Río E, Ramos-Villagrasa PJ, Castro Á, Barrada JR (2019). Sociosexuality and bright and dark personality: The prediction of behavior, attitude, and desire to engage in casual sex. International Journal of Environmental Research and Public Health.

[CR18] Fisher ML, Worth K, Garcia JR, Meredith T (2012). Feelings of regret following uncommitted sexual encounters in Canadian university students. Culture, Health & Sexuality.

[CR19] Flack WF, Hansen BE, Hopper AB, Bryant LA, Lang KW, Massa AA, Whalen JE (2016). Some types of hookups may be riskier than others for campus sexual assault. Psychological Trauma: Theory, Research, Practice, and Policy.

[CR20] Galperin A, Haselton MG, Frederick DA, Poore J, von Hippel W, Buss DM, Gonzaga GC (2013). Sexual regret: Evidence for evolved sex differences. Archives of Sexual Behavior.

[CR21] Gleason N, Banik S, Braverman J, Coleman E (2021). The Impact of the COVID-19 pandemic on sexual behaviors: Findings from a National survey in the United States. The Journal of Sexual Medicine.

[CR22] Gray JA (1970). The psychophysiological basis of introversion-extroversion. Behavior Research and Therapy.

[CR23] Gray JA (1987). Perspectives on anxiety and impulsivity: A commentary. Journal of Research in Personality.

[CR24] Grubbs JB, Wright PJ, Braden AL, Wilt JA, Kraus SW (2019). Internet pornography use and sexual motivation: A systematic review and integration. Annals of the International Communication Association.

[CR25] Heldman C, Wade L (2010). Hook-up culture: Setting a new research agenda. Sexuality Research and Social Policy.

[CR26] Kaiser HF (1974). An index of factorial simplicity. Psychometrika.

[CR27] Kennair LEO, Wyckoff JP, Asao K, Buss DM, Bendixen M (2018). Why do women regret casual sex more than men do?. Personality and Individual Differences.

[CR28] Klasen S, Schüler D (2011). Reforming the gender-related development index and the gender empowerment measure: Implementing some specific proposals. Feminist Economics.

[CR29] Le Gall A, Mullet E, Shafighi SR (2002). Age, religious beliefs, and sexual attitudes. Journal of Sex Research.

[CR30] LeFebvre LE (2018). Swiping me off my feet: Explicating relationship initiation on Tinder. Journal of Social and Personal Relationships.

[CR31] Lehmiller JJ, Garcia JR, Gesselman AN, Mark KP (2021). Less sex, but more sexual diversity: Changes in sexual behavior during the COVID-19 Coronavirus pandemic. Leisure Sciences.

[CR32] Lyons HA, Manning WD, Longmore MA, Giordano PC (2014). Young adult casual sexual behavior: Life-course-specific motivations and consequences. Sociological Perspectives.

[CR33] Madkan VK, Giancola AA, Sra KK, Tyring SK (2006). Sex differences in the transmission, prevention, and disease manifestations of sexually transmitted diseases. Archives of Dermatology.

[CR34] Maticka-Tyndale E, Herold ES, Oppermann M (2003). Casual sex among Australian schoolies. Journal of Sex Research.

[CR35] McMahan KD, Olmstead SB (2021). Are college students’ perceptions of the developmental features of emerging adulthood associated with motivations for sex? Implications for research and policy. Sexuality Research and Social Policy.

[CR36] Meston CM, Buss DM (2007). Why humans have sex. Archives of Sexual Behavior.

[CR37] Monto MA, Carey AG (2014). A new standard of sexual behavior? Are claims associated with the “hookup culture” supported by general social survey data?. The Journal of Sex Research.

[CR38] Napper LE, Montes KS, Kenney SR, LaBrie JW (2016). Assessing the personal negative impacts of hooking up experienced by college students: Gender differences and mental health. The Journal of Sex Research.

[CR39] Owen J, Fincham FD, Moore J (2011). Short-term prospective study of hooking up among college students. Archives of Sexual Behavior.

[CR40] Paul EL, McManus B, Hayes A (2000). “Hookups”: Characteristics and correlates of college students' spontaneous and anonymous sexual experiences. Journal of Sex Research.

[CR41] Pickel KL, Gentry RH (2017). Slut shaming in a school bullying case: Evaluators ignore level of harm when the victim self-presents as sexually available. Sex Roles.

[CR42] Pillsworth EG, Haselton MG, Buss DM (2004). Ovulatory shifts in female sexual desire. Journal of Sex Research.

[CR43] Ranzini G, Lutz C (2016). Love at first swipe? Explaining Tinder self-presentation and motives. Mobile Media & Communication.

[CR44] Rosenberg M, Luetke M, Hensel D, Kianersi S, Fu TC, Herbenick D (2020). Depression and loneliness during COVID-19 restrictions in the United States, and their associations with frequency of social and sexual connections. MedRxiv.

[CR45] Sevi B, Aral T, Eskenazi T (2018). Exploring the hook-up app: Low sexual disgust and high sociosexuality predict motivation to use Tinder for casual sex. Personality and Individual Differences.

[CR46] Stark R, Kagerer S, Walter B, Vaitl D, Klucken T, Wehrum-Osinsky S (2015). Trait sexual motivation questionnaire: Concept and validation. The Journal of Sexual Medicine.

[CR47] Sumter SR, Vandenbosch L, Ligtenberg L (2017). Love me Tinder: Untangling emerging adults’ motivations for using the dating application Tinder. Telematics and Informatics.

[CR48] Tabachnick BG, Fidell LS (2007). Using multivariate statistics.

[CR49] Townsend JM, Wasserman TH (2011). Sexual hookups among college students: Sex differences in emotional reactions. Archives of Sexual Behavior.

[CR50] Townshend JM, Kambouropoulos N, Griffin A, Hunt FJ, Milani RM (2014). Binge drinking, reflection impulsivity, and unplanned sexual behavior: Impaired decision-making in young social drinkers. Alcoholism: Clinical and Experimental Research.

[CR51] Trivers, R. (1972). Parental investment and sexual selection. Sexual Selection & the Descent of Man, Aldine de Gruyter, New York, pp. 136–179. http://joelvelasco.net/teaching/3330/trivers72-parentalinvestment.pdf

[CR52] Twenge JM, Sherman RA, Wells BE (2017). Declines in sexual frequency among American adults, 1989–2014. Archives of Sexual Behavior.

[CR53] Uecker JE, Martinez BC (2017). When and why women regret sex in hookups more than men do: An analysis of the online college social life survey. The Sociological Quarterly.

[CR54] Ueda P, Mercer CH, Ghaznavi C, Herbenick D (2020). Trends in frequency of sexual activity and number of sexual partners among adults aged 18 to 44 years in the US, 2000–2018. JAMA Network Open.

[CR55] Vrangalova Z (2015). Does casual sex harm college students’ well-being? A longitudinal investigation of the role of motivation. Archives of Sexual Behavior.

[CR56] Vrangalova Z, Ong AD (2014). Who benefits from casual sex? The moderating role of sociosexuality. Social Psychological and Personality Science.

[CR57] Wellings K, Palmer MJ, Machiyama K, Slaymaker E (2019). Changes in, and factors associated with, frequency of sex in Britain: Evidence from three national surveys of sexual attitudes and lifestyles (Natsal). British Medical Journal.

[CR58] Wesche R, Claxton SE, Waterman EA (2020). Emotional outcomes of casual sexual relationships and experiences: A systematic review. The Journal of Sex Research.

[CR59] Wilson EC, Garofalo R, Harris DR, Belzer M (2010). Sexual risk taking among transgender male-to-female youths with different partner types. American Journal of Public Health.

